# Disseminated Bartonellosis Masquerading as Autoimmune Glomerulonephritis: A Case Report

**DOI:** 10.1155/crin/7727894

**Published:** 2026-02-11

**Authors:** Tim Debyser, Mattias Falter, Thomas Vanhoutte, Priyanka Koshy, Melissa Depypere, Katrien De Vusser, Liesbet Henckaerts

**Affiliations:** ^1^ Department of General Internal Medicine, University Hospitals Leuven, Leuven, Belgium, uzleuven.be; ^2^ Department of Nephrology, University Hospitals Leuven, Leuven, Belgium, uzleuven.be; ^3^ Department of Microbiology, Immunology and Transplantation, KU Leuven, Leuven, Belgium, kuleuven.be; ^4^ Department of Pathology, University Hospitals Leuven, Leuven, Belgium, uzleuven.be; ^5^ Department of Laboratory Medicine, University Hospitals Leuven, Leuven, Belgium, uzleuven.be

**Keywords:** *Bartonella henselae*, case report, endocarditis, glomerulonephritis, vasculitis

## Abstract

**Background:**

Although *Bartonella henselae* is primarily known for causing self‐limiting cat scratch disease in immunocompetent individuals, it can also lead to severe disseminated infections, particularly in immunocompromised patients.

**Case Presentation:**

We present a rare case of disseminated *B. henselae* infection in a 70‐year‐old man with multiple comorbidities, including a recent aortic valve replacement and pacemaker implantation. The patient initially presented with purpuric skin lesions, progressive renal impairment, and pancytopenia, leading to a preliminary diagnosis of autoimmune glomerulonephritis and treatment with immunosuppressants. Subsequent investigations, including kidney and skin biopsies, revealed C3‐dominant glomerulonephritis and leukocytoclastic vasculitis, respectively. Further imaging uncovered a concurrent lung malignancy, treated with radiotherapy. Five months later, the patient presented with blood culture–negative endocarditis complicated by septic embolic strokes, persisting pancytopenia, and hepatosplenomegaly. Serology and *B. henselae* PCR of bone marrow confirmed disseminated *Bartonella henselae* infection.

**Conclusions:**

This case highlights the diagnostic challenges of disseminated *Bartonella* infections, which can mimic autoimmune diseases and delay appropriate treatment. Clinicians should maintain a high index of suspicion to ensure timely diagnosis and management.

## 1. Introduction

*Bartonella* spp. are small fastidious Gram‐negative​ bacilli known to cause infections in both immunocompetent and immunocompromised patients. *Bartonella henselae* typically causes cat scratch disease (CSD), a self‐limiting and potentially suppurative regional lymphadenopathy with fever and malaise after being scratched or bitten by an infected cat [1, 2]. In contrast, immunocompromised patients may develop a wide spectrum of severe clinical manifestations in several organs, such as bacillary angiomatosis and peliosis, blood culture–negative infective endocarditis, osteomyelitis, meningoencephalitis, and retinal involvement [3, 4].

Here, we present a rare case of disseminated *B. henselae* infection masquerading as autoimmune glomerulonephritis (GN), with endocarditis, cutaneous vasculitis, and bone marrow invasion.

## 2. Case Presentation

A 70‐year‐old man presented to the emergency department with a 2‐week history of general malaise and fever, associated with increased inflammatory markers. His medical history included Type 2 diabetes mellitus, alcohol use disorder, and surgically cured malignant melanoma. Nine months earlier, he had undergone aortic valve replacement with a bioprosthesis for severe aortic stenosis, followed by pacemaker implantation for symptomatic second‐degree atrioventricular block. At the time of admission, he was on mycophenolic acid 500 mg twice daily and methylprednisolone 2 mg daily for immune‐mediated paraneoplastic kidney disease.

The immunosuppressive regimen had been initiated during a prior hospitalization several months earlier, when he presented with purpuric lesions on the legs, severe kidney function impairment, and pancytopenia. At that time, skin biopsy revealed leukocytoclastic vasculitis, and serology demonstrated p‐ANCA positivity with elevated Proteinase 3 antibodies. Kidney biopsy showed focal proliferative glomerulonephritis (GN) with C3‐dominant staining, with early cellular crescent formation (Figure 1). In the setting of positive ANCA, proven cutaneous vasculitis, and kidney function impairment, rapidly progressive ANCA‐associated vasculitis was suspected and immunosuppressants were initiated (corticosteroid monotherapy followed by combination therapy with mycophenolic acid). Whole‐body FDG‐PET/CT at that time revealed a hypermetabolic hilar lymph node, which was confirmed to be a metastasis of a squamous cell carcinoma of the lung by biopsy, and stereotactic radiotherapy was commenced. Extensive investigations (including a workup for endocarditis) did not reveal an infectious cause, and immunosuppressive therapy for the suspected autoimmune or cancer‐related vasculitis was continued.

FIGURE 1Formalin‐fixed, paraffin‐embedded kidney tissue sections were stained (and scored on a scale of 0–3) for IgG (0/3), IgM (trace to 1+), IgA (trace), C3 (2 to 3/3), C1q (1 to 2/3), kappa light chains (0/3), and lambda light chains (0/3) with diffuse granular positivity in the mesangial region segmentally along the glomerular capillary wall and negative along the tubular basement membranes and blood vessels. There was early cellular crescent formation (1 of 6 open glomeruli), and there were only minimal chronic changes. (a) Kidney biopsy with Jones silver stain showing a glomerulus with cellular crescent formation. (b) Direct immunofluorescence with C3 staining showing diffuse granular positivity in the mesangial region segmentally along the glomerular capillary wall.

**Figure:**
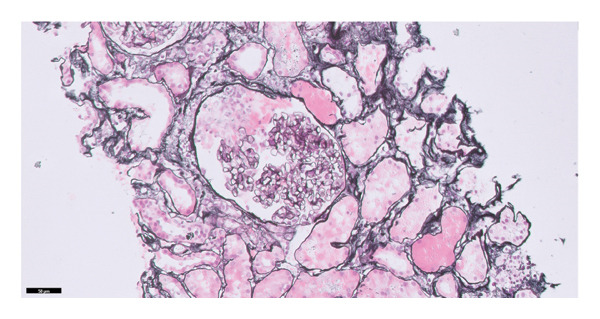
(a)

**Figure:**
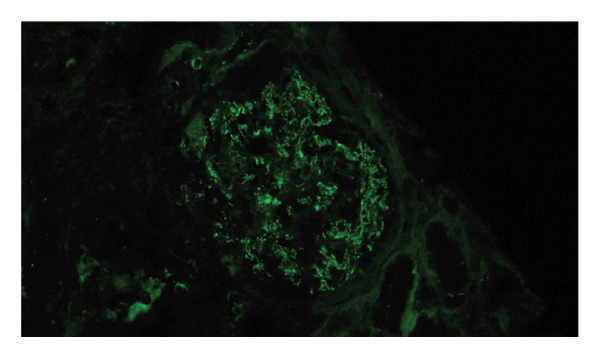
(b)

With the current presentation, the patient was hospitalized, while the initial workup (including abdominal and chest imaging, numerous blood cultures, and a urine culture) was negative. One day after admission, the patient developed a paresis of his right arm. Magnetic resonance imaging (MRI) of the brain showed recent ischemia in four separate sites, cerebral, cerebellar, as well as in the brainstem, suspicious for an emboligenic origin. A lumbar puncture was negative (no cytosis, negative Gram stain and culture, and negative multiplex PCR panel). Transesophageal echocardiography showed thickened valve leaflets of the aortic bioprosthesis with reduced motion, a new moderate aortic stenosis, and a new mild aortic valve regurgitation (three findings absent on recent transthoracic ultrasounds), but no obvious vegetations on the valves or the pacemaker leads. Repeat transesophageal ultrasound after 2 weeks showed unchanged findings. A new PET/CT scan showed hypermetabolism of the bone marrow and spleen, and moderately hypermetabolic mediastinal and bilateral hilar lymph nodes, but did not show focal uptake around the cardiac valves.

Based on these findings, culture‐negative prosthetic valve endocarditis was suspected and serological screening was performed. Serology revealed positive IgG titers (≥ 1/1280) for *Bartonella henselae*. Based on the 2023 Duke–International Society for Cardiovascular Infectious Diseases (ISCVID) Criteria (1 major microbiologic criterion and 6 minor criteria) [5], a diagnosis of definite endocarditis was made. Moreover, *B. henselae* PCR was positive on bone marrow biopsy, explaining the persisting pancytopenia and confirming active disseminated bartonellosis. Fine‐needle aspiration of an FDG–hypermetabolic subcarinal lymph node showed no signs of malignancy and showed a negative *B. henselae* PCR. Upon diagnosis, the patient was commenced on doxycycline 100 mg twice daily in association with rifampicin 300 mg twice daily.

We requested *B. henselae* serology on a stored serum sample from 5 months prior, which was found to be positive with elevated IgG (≥ 1/1280) and IgM titers (≥ 1/100). This indicates that the *Bartonella* infection was already present, and it was the likely cause of the GN, although other signs of endocarditis were still absent at that time.

Regrettably, the patient died unexpectedly after an in‐hospital cardiac arrest on Antibiotic treatment day 14. The exact cause of death was unknown; an autopsy was not performed.

## 3. Discussion

*Bartonella henselae* is a well‐established but rare cause of blood culture–negative infective endocarditis (IE) [6], with *B. quintana* and *B. henselae* causing the majority of cases [7]. In our case, the diagnosis of *Bartonella* IE was only established once *Bartonella* serology was obtained, several months after the first presentation. The observed diagnostic and therapeutic delay is not uncommon and reflects the fact that *Bartonella* endocarditis can escape early recognition, particularly when the clinical picture overlaps with autoimmune disease. *Bartonella* spp. have been implicated in up to 28% of blood culture–negative IE cases [8–11]. Patients with *Bartonella* IE are far more likely to develop GN than when IE is caused by other bacteria [12]. In a recent study on IE, *Bartonella* infection was associated with GN with an odds ratio of 38.2 (95% confidence interval: 6.7–718.8), whereas other bacteria were not [12]. In that study, 8 of 9 patients (89%) with *Bartonella* IE had GN. In another case series of 24 *Bartonella* IE cases, 21 (88%) had GN [13]. *Bartonella*‐related GN shares laboratory and biopsy features with autoimmune diseases (especially with PR3‐ANCA positivity), often leading to initial diagnostic misclassifications [13–18] and treatment with immunosuppression in up to 55% of cases [19]. Several case reports have reported various morphological presentations of *B. henselae* endocarditis–associated GN, though studies investigating the true prevalence of renal presentation forms are lacking [20]. In our case, the absence of evidence for infection led to the initial diagnosis of an autoimmune or paraneoplastic C3‐dominant GN, although the findings of a mildly elevated C3 nephritic factor and low‐grade polyclonal cryoglobulinemia probably reflected chronic infection–related inflammation rather than primary autoimmune phenomena [18, 21]. Importantly, at the first presentation, no evidence for endocarditis was observed. This is concordant with reported cases where autoimmune disease is suspected, and a new diagnostic cascade is triggered after the development of cardiac valve abnormalities [22].

Hematological abnormalities such as anemia and thrombocytopenia have been reported in *Bartonella* endocarditis [8] and are observed more frequently in a subset of patients with *Bartonella*‐associated GN, compared to patients with blood culture–positive endocarditis‐related GN [19]. Pancytopenia due to bacterial invasion of the bone marrow, as in our case, has only rarely been described [23, 24]. Moreover, our patient fulfilled the HLH‐2004 diagnostic criteria for reactive hemophagocytosis [25], which has also been described [23, 26].

The diagnosis of *Bartonella* infection mainly relies on serology usually by indirect fluorescent antibody (IFA) assays, although several limitations exist such as limited sensitivity of especially IgM antibody tests [27, 28], limited specificity of IgG antibody tests due to cross‐reactivity with other pathogens [6], and potentially high seroprevalence in the healthy population, ranging from 3.6% to 23.5% [27, 29–34]. Moreover, the sensitivity of IFA IgG tests in *Bartonella* spp. endocarditis remains suboptimal, particularly at the cutoff titer of ≥ 1/800 described by the 2023 Duke–ISCVID Criteria, potentially missing patients with a lower antibody titer [5, 6, 35]. PCR on affected solid tissue, such as valve tissue, shows higher sensitivity than serology tests [6], though it involves more invasive testing. PCR on blood may offer an alternative, although this has a low sensitivity in patients with CSD [36] and *Bartonella* spp. endocarditis [6]. Metagenomic (“shotgun”) sequencing is another promising diagnostic tool for blood and valve tissue [37–39], although not readily available in all centers. In our case, *B. henselae* IgM and IgG antibodies by IFA and *B. henselae* PCR on bone marrow tissue were positive.

The most recent ESC Guidelines recommend combination therapy with oral doxycycline (4 weeks) and intravenous gentamicin (2 weeks) for the treatment of *Bartonella* IE [40]. We chose to replace gentamicin with rifampicin because of his precarious kidney function [41]. Unfortunately, we could not evaluate the effect of the antibiotic therapy due to the premature death of our patient.

We conclude that disseminated *Bartonella* infection poses a diagnostic challenge, often masquerading as autoimmune or systemic diseases. This case underscores that clinicians should be vigilant for *Bartonella henselae* infections beyond the classical CSD presentation. Importantly, a negative animal exposure history does not exclude *Bartonella* infection due to the possibility of unrecognized transmission routes and the limitations of patient recall. Similarly, *Bartonella quintana* infections can also be associated with immunological abnormalities such as GN and ANCA positivity [42] and must be considered even in the absence of typical risk factors such as homelessness [43]. Systematic exclusion of underlying infectious causes before initiating immunosuppressive therapy in patients with GN and an atypical clinical picture is thus critically important. *Bartonella* infection should therefore be actively considered in selected clinical presentations. In our opinion, these include patients with culture‐negative endocarditis, unexplained systemic inflammatory syndromes (e.g., with fever, lymphadenopathy, or splenomegaly), unexplained cytopenias (especially in patients with a prosthetic valve), cases of GN with unusual immune profiles (such as C3‐dominant GN, polyclonal cryoglobulinemia, or ANCA positivity), and those with suspected autoimmune disease demonstrating an atypical or insufficient response to immunosuppressive treatment. Incorporating *Bartonella* serology into the diagnostic workup in these scenarios may help prevent misdiagnosis, avoid inappropriate immunosuppressive treatment, and enable timely initiation of targeted antimicrobial therapy.

## Funding

No funding was received for this case report.

## Ethics Statement

The Ethics Committee of the University Hospitals Leuven approved the writing of this case report (S68992). The Ethics Committee granted a waiver for informed consent, as the patient had unfortunately passed away and could not give consent. This case report was conducted in accordance with the principles of the Declaration of Helsinki.

## Consent

Please see the Ethics Statement.

## Conflicts of Interest

The authors declare no conflicts of interest.

## Data Availability

The data that support the findings of this study are available on request from the corresponding author. The data are not publicly available due to privacy or ethical restrictions.
